# Applied insights for using Generative Artificial Intelligence in Faculty Development in Health Professions Education

**DOI:** 10.12688/mep.21403.1

**Published:** 2025-12-08

**Authors:** Melchor Sánchez-Mendiola, Megan Anakin, Ardi Findyartini, Rachel Levine, Ana Da Silva, Farhan Saeed Vakani

**Affiliations:** 1 Postgraduate Studies Division, National Autonomous University of Mexico Faculty of Medicine, Mexico City, Mexico City, 04510, Mexico; 2 Pharmacy Education, The University of Sydney School of Pharmacy, Sydney, New South Wales, 2006, Australia; 3 Indonesia Medical Education and Research Institute, University of Indonesia Faculty of Medicine, Jakarta, Special Capital Region of Jakarta, 16424, Indonesia; 4 Faculty Development Program, Johns Hopkins University School of Medicine, Baltimore, Maryland, 21205, USA; 5 New Medical Education programmes, Swansea University Medical School, Swansea, Wales, SA2 8PP, UK; 6 Dow Institute of Health Professionals Education, Dow University of Health Sciences, Karachi, Sindh, 74200, Pakistan

**Keywords:** Generative artificial intelligence, Faculty development, Health professions education, Ethics, Equity, Technology-enhanced learning

## Abstract

**Introduction:**

Generative AI (GenAI) tools are transforming health professions education, offering opportunities to enhance faculty development (FD). Faculty developers are uniquely positioned to integrate GenAI into practice to address resource constraints, improve accessibility, and foster equity across diverse educational contexts. This Applied Insights article offers a perspective on how GenAI can be leveraged as a co-developer in FD by drawing on emerging literature and discussion points from a workshop at the *8th International Faculty Development Conference in the Health Professions*.

**Applied insights:**

The applied insights are structured around key phases of FD: planning, content creation, delivery, and evaluation. They include actionable strategies for using GenAI in needs assessment, multilingual and culturally relevant resource creation, personalized learning plans, and when providing feedback and mentorship. Each insight is rooted in pedagogical rationale, evidence, and strategies to address ethical and practical challenges, with an emphasis on human oversight, contextual relevance, and continuous evaluation of GenAI’s impact.

**Conclusions:**

By considering these insights, faculty developers can harness GenAI to co-design educational materials, extend their reach through innovative formats, and maintain ethical and equity-driven educational practices. This article highlights the transformative potential of GenAI in FD when thoughtfully integrated. GenAI can empower faculty developers to enhance the quality and inclusivity of HPE while safeguarding educational standards.

## Introduction

Generative AI (GenAI) has emerged as a disruptive force in health professions education, transitioning from novelty to necessity in a short time span ^1^. The integration of GenAI tools in education is accelerating, prompting urgent questions for faculty development (FD) ^2^. Globally, faculty developers face a timely imperative to understand both GenAI’s capabilities and limitations to support them through the excitement and anxiety surrounding AI ^3^. In addition to supporting staff members as educators, researchers, leaders, and administrators in health professions education, faculty developers are key agents in translating innovations into effective teaching practice ^4^.

GenAI can help bridge resource gaps across diverse settings. It enables personalized content creation and can save time and costs in generating teaching materials and assessment items, without compromising quality. GenAI supports resource development in multiple languages and cultures. This versatility may be valuable in low-resource settings that lack access to professional translation or content experts. GenAI offers ways to scale and tailor FD offerings to local needs, aligning with principles of equity and contextual relevance ^5^.

The adoption of GenAI brings challenges. There is a tension between innovation and responsible use. This tension can produce concerns about ethics, bias, academic integrity, and equity. Faculty developers must manage questions of accuracy, privacy, and fairness in GenAI content, ensuring that the drive to innovate does not exacerbate existing disparities. Staff members may be unsure how to effectively use GenAI tools ^6^. This gap in understanding highlights an opportunity for faculty developers to build GenAI capabilities with their colleagues.

This Applied Insights article focuses on how faculty developers are using GenAI in their work settings, rather than merely teaching others about it. The intent is to empower faculty developers as responsible users of GenAI by integrating these tools as partners in designing, implementing, and evaluating FD programs. The following insights provide a practical roadmap. They include evidence from emerging literature and illustrative scenarios drawn from personal experience in a faculty development workshop at the *8th International Conference on Faculty Development in the Health Professions* (ICFDHP). Each applied insight addresses challenges, presents ethical considerations, and suggests ideas for continuous evaluation to reinforce that human oversight and critical judgment are essential as we integrate GenAI in our work.

## Applied insights

### #1. Ground your approach to using GenAI in faculty development with key principles

Key principles for GenAI use in medical education are human-centered design, transparency, contextual relevance, ethical awareness, equity, and continuous reflection ^7, 8^. An overarching theme is that the human remains the ultimate decision-maker ^5^. Faculty developers need to consider if the data will be used to train the GenAI tool, construct new models, or to generate new content for other users. Prompts using student and patient information should be avoided because even if it is anonymized; the information entered may be stored and used for other purposes. Faculty developers must assist staff members to become aware of the data implications of using GenAI and role model its use following local institutional guidelines.

GenAI models can reproduce or amplify biases present in their training data. In healthcare education, these biases could mean stereotyping roles, underrepresenting certain populations, or providing data reflecting the dominant culture on which the model is based. To address biases in GenAI outputs, faculty developers and staff members can actively engage in identifying and addressing bias, a critical component of AI literacy and ethical awareness.

Using only institutionally approved or privacy-compliant tools such as ChatGPT Enterprise, Claude 3, or Perplexity Pro, helps to maintain confidentiality. Before any session, faculty developers should design and use a GenAI checklist to clarify whether data are sensitive, determine how accuracy will be verified, and identify who will review the final content. Checking for accuracy and bias should be a routine, structured step rather than an afterthought. A practical workflow can be implemented with two passes, for accuracy and bias. The accuracy pass focuses on checking factual claims and citations using reliable databases or PubMed searches. This pass involves comparing multiple GenAI outputs (ChatGPT, Claude, Perplexity) to identify inconsistencies. The bias pass involves conducting a representation audit. Begin the audit by prompting the GenAI to critique its own output (e.g., Identify any gender, cultural, or disciplinary biases in this text) and also review output by looking for stereotypes or omissions. Tools such as Biasly AI ( https://www.biasly.com) can assist in flagging imbalanced language.

The bias pass also requires another person to check that the GenAI output makes sense from their perspective. Invite a colleague from a different discipline, gender, or region to review the material for tone and inclusivity.

Documenting these reviews in a GenAI-use log improves transparency and provides valuable evidence for quality assurance and scholarly reflection. By demonstrating how to question GenAI outputs for accuracy and bias, faculty developers normalize critical engagement rather than passive adoption.

### #2. Use GenAI-supported needs analysis

Faculty developers can use GenAI to gather information for planning and discussion in FD sessions. By reflecting on needs assessment data together, staff members' concerns can be validated and set the stage for a learner-centred activity. GenAI can analyze information provided by staff members to quickly identify common gaps in skills or knowledge ^9^. For example, anonymized comments from faculty surveys can be imported into ChatGPT or Claude with this prompt: *‘*Summarize the recurring challenges these educators face in providing clinical feedback. Group them into teaching, assessment, and leadership categories.’ This summary can be created in minutes, offering a draft “diagnosis” of common needs around which to design a workshop or course. The GenAI’s summary can then be reviewed and refined collaboratively, encouraging participants to validate whether the generated themes resonate with their lived experiences.

Faculty developers must still act as interpreters, not consumers, of GenAI analysis. Cross-checking with alternative data sources or discussing the GenAI-derived summary in small groups enhances validity and transparency. In practice, the combination of machine synthesis and human interpretation yields a richer, more inclusive understanding of faculty needs.

### #3. Teach prompt literacy through cognitive apprenticeship

The ability to craft effective prompts is known as prompt literacy, and it has become a central digital competence for educators ^10^. Faculty developers who model effective prompting help peers build confidence when using GenAI. When faculty developers verbalize prompt construction, it can improve an educator’s ability to contextualize and assess the quality of GenAI outputs.

A powerful way to teach prompt literacy involves a cognitive apprenticeship approach ^11^, where a faculty developer “thinks aloud” and demonstrates prompting in real-time. When using a GenAI tool in a workshop, faculty developers can narrate their thought process to explain how a question is refined, context is added, and parameters are adjusted to get a better output. By externalizing expert thinking, GenAI use can be demystified while the cognitive load for novices is reduced. Table 1 shows an outline of prompting principles to help make the trial-and-error process of prompt crafting explicit; this framework was developed by the authors. See the Appendix for worked-out examples and suggestions.

**Table 1.** CRAFT outline of prompting principles.

Principle	Description	Why it matters
**C – Contextualize**	Ground the prompt in institutional, cultural, or pedagogical setting.	Prevents generic, culturally irrelevant outputs.
**R – Role-switching**	Ask GenAI to adopt expert roles (mentor, reviewer, designer) or multiple viewpoints.	Encourages critical thinking, reflection, and dialogue from multiple perspectives.
**A – Augment, don’t automate**	Use GenAI to spark or scaffold rather than fully replace academic tasks.	Keeps faculty developer in the role of final editor and sense-maker.
**F – Feedback-driven refinement**	Always review and improve the prompt iteratively based on GenAI output.	GenAI improves with specificity and correction.
**T – Traceability & ethics**	Record prompts and edits. Add a disclosure if used in published or assessed work.	Supports academic integrity and replicability.

**Appendix.** Prompting CRAFT framework with examples and suggestions.

Principle	Examples and suggestions
**C – Contextualize**	Prompt - *I am designing a 3-hour faculty development workshop for medical educators at a public medical school in Mexico City. The participants will be early- and mid-career clinicians and academics who teach in Spanish, often balancing heavy clinical workloads, large classes, and limited pedagogical support.* *The faculty is seeking to modernize its teaching approaches, moving from lecture-based instruction toward competency-based and active learning methods, aligned with national accreditation standards (e.g., COMAEM) and the university’s educational model.*
**R – Role-switching**	Prompt - *You are an expert assessor and educational supervisor in health professions education in [insert country], experienced in evaluating post-graduate training portfolios. Please provide constructive, criterion-based feedback on the reflective portfolio text below, using the [insert country name] National Standard for [insert profession or level — e.g., medical education, teaching, or clinical training] as the reference framework.*
**A – Augment, don’t automate**	Suggestion - Always review carefully the outputs, ask yourself: - Are claims verifiable and current? - Does this output serve the intended purpose clearly and appropriately for the audience? - Would this be fair and inclusive in my institution’s context? - If using references, make sure you check them all and that these are being used appropriately.
**F – Feedback-driven refinement**	Suggestions - Use expressions like “make it clearer, more specific, and less ambiguous.” Identify vague terms and suggest replacements that would guide the AI toward more consistent, contextually relevant results. Optimise the prompt by defining the AI’s role, perspective, and tone more clearly (e.g., expert mentor, policy adviser, educational designer). Suggest how to phrase it to get authoritative yet supportive responses.
**T – Traceability & ethics**	Suggestions - Save chats in your account screenshots, some GenAI allows for PDF export. Others may provide a link (e.g. ChatGPT Share> copy link) to the chat that you can keep for reference/audit trail.

Faculty developers do not need to be expert users of GenAI to demonstrate prompting. They can explore the process of crafting prompts with staff members to show how critical analysis and judgement can improve it. This co-exploration showcases the iterative nature of working with GenAI and emphasizes that prompt literacy is an ongoing learning process for everyone. In workshops, participants might co-create prompts for designing a 60-minute session on feedback in clinical teaching, compare results, and discuss why certain formulations produced superior outcomes. This shared experimentation increases confidence and GenAI literacy.

Faculty developers can use tools like PromptPerfect ( https://promptperfect.xyz) to analyze prompt efficiency or explore shared examples in prompt libraries ( https://library.maastrichtuniversity.nl/apps-tools/ai-prompt-library/; https://promptathon.iime.cloud).

### #4. Co-create multilingual and context-specific resources using GenAI

Faculty developers can use GenAI to co-create multilingual and contextually relevant resources. GenAI use will support them to model culturally responsive pedagogy ^12^. By co-designing materials with GenAI, faculty developers can access content from different settings and refine it to ensure local relevance ^13^. They can use GenAI to produce an initial translation or adaptation of a resource and then add the critical layer of cultural and pedagogical quality control.

For example, materials in English can be translated and culturally adapted for use in Spanish or another language appropriate for audience members. In addition to direct translation, GenAI can suggest localized examples or metaphors that can be reviewed for appropriateness. Faculty developers can use ChatGPT, Claude or DeepL Write ( https://www.deepl.com/es/write) to translate teaching materials, ensuring contextual accuracy by specifying intended audiences. Faculty developers may need to invite a native speaker to review GenAI text for accuracy and sensitivity, to avoid misinterpretation.

### #5. Remix and adapt educational cases with GenAI to promote interprofessional education and inclusivity

A GenAI assistant can expedite the creative process by generating and remixing teaching scenarios swiftly. This approach saves time and operationalizes principles of equity and contextualization in FD content; however, the content must be reviewed before use. Faculty developers can update the case studies or scenarios used in workshops to stimulate discussion and application of concepts by GenAI to represent several health professions and the diversity of real-world practice ^14^. For example, GenAI tools like ChatGPT or DeepSeek can be used to convert a physician-centric case into an interprofessional scenario with multiple healthcare roles or shorten a lengthy case to fit a tighter timeframe.

GenAI might introduce fictional elements or errors while remixing the case. For instance, Gen AI might assign a pharmacist a responsibility they typically do not have or create an unrealistic scenario. To identify errors, double-check with subject matter experts, assess the representatives of the professions, or consult the literature on roles and scope of practice. Stereotypes can be avoided by explicitly prompting GenAI to avoid them. Faculty developers can check GenAI-generated cases for bias in simple, concrete ways. After reading the GenAI-generated text, pause to ask questions about representation, assumptions, and corrections as shown in Table 2 to ensure the material represents people and professions fairly and respectfully.

**Table 2.** Questions to check for representation and assumptions, and to make corrections.

Topic	Questions	Reason
**Representation**	Who is missing or stereotyped?	To check whether people of different genders, professions, or cultural backgrounds are shown in limited or unequal roles.
	Are some professions always in “supporting” roles while others always lead?	To identify patterns where certain groups (e.g. nurses, pharmacists) are consistently positioned as secondary or invisible.
	Do patients and families appear as active partners, or only as passive recipients?	To ensure patients and families are represented as decision-makers, not just as background figures.
	Would the case still “work” if we changed the gender, ethnicity, or socioeconomic status of the main character?	To see whether the story relies on stereotypes tied to a particular identity.
**Assumptions**	Does the case make hidden judgments?	To look for language that implies blame, heroism, or cultural superiority, such as describing a patient from one background as “noncompliant” without context.
	What is being taken for granted about this patient, team, or setting?	To reveal assumptions (e.g. that all learners have the same resources, or that one profession always has the “final say”).
	Is any group consistently blamed when things go wrong, or always praised when things go well?	To detect one-sided portrayals that may reinforce stigma or unrealistic “hero” narratives.
	Are there value-laden words (e.g. “difficult”, “demanding”, “noncompliant”) that could be replaced with more neutral descriptions?	To encourage more descriptive, less judgmental language.
**Corrections**	What stereotypes might be present?	To prompt explicit identification of clichés (e.g. male surgeon, female nurse) that can be altered.
	What perspective might be missing (patient, family, nurse, pharmacist, community)?	To remind faculty to add voices that are often overlooked but relevant to real practice.
	How could I rewrite this paragraph so that different professions contribute more equally?	To convert the bias check into a concrete edit of the text.
	What specific change will I make now (e.g. change wording, add a character, adjust who speaks)?	To ensure the bias scan results in an actual correction, not just awareness.
	Can GenAI help me revise this case to reduce bias if I ask it directly?	To encourage using GenAI itself for revision (e.g. “Rewrite this case to avoid stereotypes and distribute decision-making across team members”), followed by human review. GenAI can help you perform the accuracy and bias passes suggested in applied insight #1

### #6. Provide formative feedback on teaching practice using GenAI

GenAI can serve as a teaching coach to provide structured and rapid feedback to educators, especially when human feedback is limited. GenAI may assist educators to reflect-in-action since formative feedback that is structured and continuously available supports self-regulated learning ^15^. Getting prompt feedback on one’s teaching design or performance can lead to improvement ^16^. Additionally, emerging evidence suggests that GenAI-assisted feedback can deepen educators’ reflection ^17^. Educators can use GenAI to provide themselves with formative feedback such as pointing out unclear objectives or suggesting new resources.

GenAI also allows for immediate follow up and extension. Educators can prompt ChatGPT or Claude with questions such as ‘How could I make my slides more interactive?’. This feedback can be considered by educators independently or discussed with a faculty developer, especially if it appears to be off-target or too generic. Faculty developers can use GenAI for formative feedback on teaching artefacts, such as lesson plans, slide decks, or recorded teaching encounters. This feedback can be used as starting point for a discussion about teaching practice.

If GenAI produces feedback such as “The assessment method isn’t clear,” a faculty developer can ask an educator, “Do you feel your assessment plan is clear? Let’s see if we can refine it.” If Gen AI provides off-target advice, then the faculty developer can use it as a teaching point about GenAI’s limitations. GenAI provides immediate formative feedback, but it requires scrutiny to ensure it can be used constructively to improve practice.

### #7. Scaffold personalized professional development plans

Professional development is most effective when it is tailored to the individual’s goals and context ^18^. Staff members find the formulation of clear actionable professional development plans challenging, and GenAI can support this task. Self-determination theory suggests that individuals are more engaged when they have autonomy in setting goals and perceive that they are building competence ^19^. GenAI can assist by providing templates or first drafts of personalized development plans that a staff member can refine and customize to meet regulatory standards and supervisor expectations ^20^.

By including a staff member’s career stage, interests, and required competencies, GenAI can generate a structured development plan aligned with those parameters ^21^. Faculty developers can provide a checklist of must-haves drawn from professional standards so each person can ensure their plan includes basic requirements. Staff members may add parameters unique to their situation. For example, if they are in a rural area or have a teaching-focused role with limited research time, the plan should reflect those constraints.

If staff members share parameters, GenAI may provide similar suggestions such as to attend a conference, read specific journals, or take a particular course. Faculty developers can add prompts to include at least one novel or uncommon development activity in its list of suggestions, these can be enhanced with peer ideas. The use of GenAI to develop personalized development plans can be revised over time to reflect professional growth.

For instance, a new clinical educator might ask ChatGPT to draft a six-month plan balancing teaching innovation, scholarly writing, and leadership growth. The draft can then be refined with mentor input and tracked using platforms such as Trello ( https://trello.com). Faculty developers can facilitate workshops where participants co-create and critique GenAI-assisted plans, comparing how well the recommendations fit real-world constraints.

### #8. Evaluate continuously and iteratively

Embracing GenAI in FD should be accompanied by a commitment to continuous and iterative evaluation. Faculty developers should treat GenAI innovations as interventions to be studied and improved. They must keep FD activities human-centered and evidence-informed with GenAI as a dynamic partner that is continually shaped to serve our educational mission ^22^. This partnership means collecting data on learning gains, efficiency, and participant feedback, and using that evidence to refine how GenAI can be used in subsequent iterations. For example, ChatGPT can condense post-workshop reflections into themes such as “time savings,” “improved creativity,” or “ethical concerns.” These summaries can inform iterative improvement cycles.

Continuous evaluation also requires faculty developers to notice broader issues such as over-reliance or unequal use of GenAI among staff members. Questions to probe these issues include: ‘Are people bypassing critical thinking because GenAI gives quick answers? Are all staff members benefiting equally, or are there groups who struggle with the technology?’ Monitoring these issues will help faculty developers to ensure GenAI remains a tool that augments human capability and does not inadvertently diminish skills or exacerbate inequities.

Documenting these evaluations also contributes to the scholarship of teaching and learning. Faculty developers can treat each applied insight as an opportunity to generate hypotheses in need of validation. By collecting and responding to feedback and outcome measures, faculty developers can model reflective practice and improvement to colleagues.

## Conclusion

These practical insights offer a roadmap for embracing GenAI as a powerful adjunct to, not a replacement for, the expertise and creativity of faculty developers. A unifying theme is the principle of the human-in-the-loop: no matter how sophisticated GenAI tools become, it is the faculty developer’s judgment, ethics, and contextual understanding that direct the use of GenAI as a co-partner. GenAI can expand what is possible by generating content at scale, providing instant feedback, translating and tailoring materials. Yet, faculty developers must decide how such possibilities align with educational values and goals. The global FD community stands to gain from GenAI when it is applied judiciously. GenAI can help address equity and access issues to help faculty developers offer more inclusive interprofessional development. However, we must remain vigilant, so GenAI use reinforces humanistic, equity-driven education. The role of faculty developer has become more important than ever to critically evaluate how GenAI can act as a co-partner. It is faculty developers who ultimately provide wisdom, experience, and mentorship to guide FD in the new era of GenAI.

## Data Availability

No data are associated with this article.
