# Applied insights for using Generative Artificial Intelligence in Faculty Development in Health Professions Education

**DOI:** 10.12688/mep.21403.2

**Published:** 2026-06-15

**Authors:** Melchor Sánchez-Mendiola, Megan Anakin, Ardi Findyartini, Rachel Levine, Ana Da Silva, Farhan Saeed Vakani

**Affiliations:** 1 Postgraduate Studies Division, National Autonomous University of Mexico Faculty of Medicine, Mexico City, Mexico City, 04510, Mexico; 2 Pharmacy Education, The University of Sydney School of Pharmacy, Sydney, New South Wales, 2006, Australia; 3 Indonesia Medical Education and Research Institute, University of Indonesia Faculty of Medicine, Jakarta, Special Capital Region of Jakarta, 16424, Indonesia; 4 Faculty Development Program, Johns Hopkins University School of Medicine, Baltimore, Maryland, 21205, USA; 5 New Medical Education programmes, Swansea University Medical School, Swansea, Wales, SA2 8PP, UK; 6 Dow Institute of Health Professionals Education, Dow University of Health Sciences, Karachi, Sindh, 74200, Pakistan

**Keywords:** Generative artificial intelligence, Faculty development, Health professions education, Ethics, Equity, Technology-enhanced learning

## Abstract

**Introduction:**

Generative artificial intelligence (GenAI) has rapidly become accessible to health professions educators and learners, creating both opportunities and new responsibilities for faculty development (FD). GenAI can help draft, adapt, and translate educational materials, and can assist faculty developers as a “co-developer.” However, GenAI outputs require human oversight, and FD leaders must actively manage accuracy, privacy/confidentiality, equity/fairness, and sustainability concerns. This article offers a perspective on how GenAI can be leveraged as a co-developer in FD by drawing on emerging literature and discussion points from a workshop at the *8th International Faculty Development Conference in the Health Professions*.

**Applied insights:**

We provide eight applied insights, organised across key phases of FD work (planning, content creation, delivery, and evaluation). These insights highlight how faculty developers can: ^1^ ground GenAI use in explicit principles and governance; ^2^ use GenAI to synthesise needs assessment data; ^3^ teach prompt literacy through cognitive apprenticeship using a practical prompt framework (CRAFT); ^4^ co-create multilingual and context-specific resources; ^5^ remix and adapt teaching cases to support interprofessional learning while protecting confidentiality and addressing bias; ^6^ support feedback and mentorship with GenAI (including iterative prompting and retrieval-augmented approaches); ^7^ scaffold personalised continuing professional development plans aligned with self-determination theory; and ^8^ evaluate GenAI use continuously and iteratively. A longitudinal interprofessional vignette is woven across the paper to demonstrate how each insight can be applied in practice.

**Conclusions:**

When used responsibly, GenAI can augment FD and help educators work more efficiently without reducing the central role of human judgment, ethics, and contextual expertise. Faculty developers can increase benefits and reduce harms by making their governance explicit, modelling prompt literacy, and embedding quality assurance/quality improvement cycles.

## Introduction

Generative artificial intelligence (GenAI) is increasingly embedded in everyday educational work. GenAI can help educators when drafting cases, creating rubrics, translating materials, and generating feedback prompts. ^1^ The speed of adoption has raised questions about whether GenAI is a passing ‘fad’ or a durable shift that requires intentional faculty development (FD) responses. ^2^ ^,^ ^3^ In health professions education (HPE), the pace of change is particularly high because educators and faculty developers must balance innovation with patient safety, professionalism, and equity. ^4^

While early reports suggest that GenAI can support efficiency in educational design and delivery, it rarely produces a “final product” without human oversight. ^1^ ^,^ ^3^ GenAI outputs can be incomplete, inaccurate, culturally misaligned, or inadvertently biased. The practical challenge for FD leaders is therefore not whether to use GenAI, but how to integrate it safely and transparently into educator workflows. ^5^ ^,^ ^6^

FD is a relational and context-dependent practice that spans needs assessment, programme design, facilitation, coaching, and evaluation. ^4^ Faculty developers are well positioned to help educators move from basic awareness of GenAI to responsible, competent, and reflective use. ^6^ However, many educators report uncertainty about what GenAI can and cannot do, and they may lack confidence or shared norms for appropriate use. ^3^

In this article, we focus on using GenAI as a co-developer: a tool that can help educators think, draft, adapt, and test ideas; while educators remain accountable for decisions. We also take a “human-in-the-loop” position: ^3^ GenAI can assist but should not replace human judgment in high-stakes educational decisions, especially when assessment and progression decisions are involved.

Responsible GenAI adoption in FD must address multiple domains, including: (a) accuracy and evidence to assist with verification and citation practices, (b) privacy, confidentiality, and intellectual property including data sovereignty considerations, (c) fairness and inclusion bymitigating bias in outputs and ensuring equitable access to GenAI-enabled opportunities, and (d) sustainability by recognising that GenAI use has environmental and financial costs. ^7^ ^,^ ^8^

The eight applied insights below were developed through the authors’ faculty development practice at different institutions and through their reflections afteran interactive GenAI-focused FD workshop delivered at the 8th International Conference on Faculty Development in the Health Professions. No identifiable participant data were collected or analysed for research purposes; this article is presented as practice-based guidance rather than as a formal qualitative study. To strengthen applicability, we include a single fictional but plausible longitudinal, interprofessional vignette revisited across the eight insights.

## Approach and conceptual framing

To avoid treating GenAI as a neutral addition to our practice, we use an entangled pedagogy lens: pedagogical decisions, technologies, institutional policies, and professional values co-constitute how GenAI is actually used in practice. ^9^ GenAI is not simply a tool educators apply to teaching; it becomes part of the learning environment that shapes what educators and learners can do, notice, and value.

We also draw on AI literacy frameworks in higher education to foreground that GenAI use not only involves skills, such as writing well-constructed prompts, but also requires critical evaluation, ethical reasoning, and contextual judgement. In this article, we use two operational definitions. ^10^ ^–^ ^12^

• **Prompt literacy:** the ability to purposefully craft, test, critique, and refine prompts with their associated workflows, to obtain useful outputs, while recognising limitations and risks.
• **GenAI checklist:** a short set of pre- and post-use questions used to reduce avoidable harms, such as privacy breaches, bias, or unverified claims, and to document decisions for transparency and improvement.

Finally, we align the insights with a pragmatic educational design cycle used in FD work. This cycle involves analysing needs, designing and developing resources, enacting and coaching in context, and evaluating/iterating. This framing helps readers see how the eight insights connect into a coherent workflow rather than a standalone list of tips.

Box 1. **Longitudinal vignette: the “Feedback for Learning” interprofessional FD programme.**

Riverview Faculty of Health Sciences (a fictional, composite institution) is launching a 10-week hybrid FD programme called “Feedback for Learning.” The programme targets clinical educators from medicine, nursing, pharmacy, physiotherapy, and social work across two campuses. The immediate driver is an upcoming accreditation review requiring clearer assessment plans and more equitable feedback practices. The FD team has limited design capacity, a bilingual audience (Spanish and English), and a commitment to represent local communities, including rural, migrant, and Indigenous groups. Institutional guidance permits GenAI use for low-risk drafting tasks if no sensitive data are entered and if outputs are reviewed before use. Across the eight insights, we revisit this vignette to show how a faculty developer might use GenAI to: clarify principles and governance; analyse needs assessment data; teach prompt literacy; translate and localise materials; generate and audit interprofessional cases; support coaching conversations; scaffold individual development plans; and evaluate the programme iteratively.

## Applied insights

### #1. Ground your approach to using GenAI in faculty development with key principles

Start by explicitly stating the principles that will govern GenAI use in your FD context. This statement can reduce hidden curriculum effects, such as acknowledging that informal norms can normalise unsafe or inequitable GenAI use, and provide a shared basis for discussion. Across contexts, five principles recur in the literature and in practice: human accountability, privacy/confidentiality, fairness/inclusion, transparency, and continuous improvement. ^7^ ^,^ ^8^ We add a sixth principle: sustainability, because GenAI use has environmental and financial costs. ^13^

These principles are actionable when translated into workflow steps. We recommend two practical steps: an ‘accuracy pass’ and a ‘bias and harm pass’ for any GenAI-assisted output intended for learners or educators. The accuracy pass focuses on factual correctness and alignment with evidence, policies, and scope of practice. The bias and harm pass checks for representational bias, such as who is portrayed by default, stigmatising or colonial language, and potential harms to groups who are often marginalised in HPE. These harms can include race/ethnicity, language, disability, age, body size, gender identity, or socioeconomic status. When addressing representational bias, include where possible, more than one reviewer and invite perspectives from the communities portrayed in the output. ^14^ ^,^ ^15^

Because many GenAI tools may store prompts and/or employ user inputs to further train models, faculty developers should decide on how sensitive their data is and if it is acceptable to enter it. We suggest defining ‘sensitive data’ broadly to include: identifiable patient, learner, or staff information; confidential institutional documents; and culturally sensitive community information. If your institution or jurisdiction has guidance on approved tools, such as an institutionally licensed ‘GenAI tool’ or enterprise versions with stronger privacy controls, then use those by default. Where local guidance is absent, consult relevant national or international guidance and seek organisational consent before using GenAI in a faculty development session. ^8^ ^,^ ^14^

Box 2. **GenAI checklist for responsible faculty development use.**

• **Purpose and risk:** What is the task? Is it idea generation, summarising text, translating documents, designing an assessment, feedback production? What are the risks if the output is wrong or biased?
• **Data and confidentiality:** Does my prompt include any sensitive data? If yes: stop, de-identify, or move your prompt into an approved secure GenAI workflow.
• **Tool choice:** Am I using an institutionally approved GenAI tool? Do I know if prompts and outputs are retained or used for model training?
• **Verification plan:** How will I verify factual claims, references, policies, and scope of practice? What parts of the GenAI output require review by a subject-matter expert?
• **Fairness and inclusion:** Who is represented, who is missing, and who is portrayed as default? Is any language stigmatising, ableist, racist, or otherwise harmful?
• **Transparency and documentation:** How will I document GenAI use? Do I have a prompt log, version record, and a process for disclosing GenAI use to learners/participants when appropriate?
• **Sustainability:** Can I achieve the same outcome with fewer prompts, a smaller model, or by reusing a prompt template?
• **Accountability:** Who is intellectually responsible forthe final output? What is the escalation plan if an error or harm is identified?

*Vignette thread (Riverview - Step 1):* Before designing the programme, the FD team drafts a one-page GenAI use statement for participants. They select an institutionally approved GenAI tool, define ‘sensitive data,’ and decide that GenAI will be used only for low-risk activities such as drafting workshop outlines, translating documents from Spanish to English and vice versa. The agree to include mandatory accuracy and bias/harm passes before disseminating the program design suggested by their GenAI tool. They also create a shared prompt log and assign responsibility for final approval to the programme lead.

### #2. Use GenAI-supported needs analysis

Needs assessment remains a foundational step in FD, yet it is often constrained by time and analytic capacity, especially when data include large volumes of open-ended text. GenAI can assist as a ‘first-pass analyst’ to rapidly summarise qualitative comments, suggest theme labels, or generate draft personas representing common educator needs. This assistance can help faculty developers move quickly to design, while reserving human effort for interpretation and decision-making. ^16^

To use GenAI responsibly in needs assessment, start by removing identifiers such as names, locations, and unique roles and aggregating data by combining comments across respondents. Then, use prompts that request transparency about uncertainty. For example, GenAI can be prompted to label outputs as high confidence versus speculative. GenAI can also be prompted to quote or reference the exact phrases that support each theme or category. Finally, GenAI-suggested output can be verified with at least one human review step. This step could involve a quick team coding check, member checking with a small educator group, or comparison with prior needs assessment cycles.

A practical prompt pattern is to ask for: (a) a concise theme list; (b) 2–3 exemplar quotes per theme; (c) ‘outlier’ or minority views that should not be lost; and (d) design implications such as which session topics or formats respond to the themes. For higher-stakes uses such as when interpreting sensitive equity concerns, consider a ‘dual-analysis’ approach: run the same de-identified dataset through GenAI twice using different prompts, and compare the themes for stability before acting on them.

*Vignette thread (Riverview - Step 2):* The FD team surveys clinical educators across professions and receives 140 open-ended responses. After de-identifying the dataset, they promptGenAI to cluster comments into themes and to provide exemplar quotes. The team then reviews the themes in a 30-minute meeting, flags a missing equity theme (access to feedback opportunities in rural rotations), and adjusts the programme to include a session on equitable workplace-based assessment.

### #3. Teach prompt literacy through cognitive apprenticeship

Prompt literacy is a core capability for educators who want to use GenAI effectively and responsibly. Rather than treating prompting as an individual skill developed through trial and error, faculty developers can teach prompt literacy through cognitive apprenticeship: model the thinking process, make decisions visible, scaffold practice, and gradually fade support as participants gain competence. ^10^ ^,^ ^17^

In FD sessions, explicitly demonstrate iterative prompting. Start by modeling a simple prompt, inspect the output critically, revise the prompt, and repeat the query sequence. This modeling allows participants to see that GenAI outputs are drafts and not final products, and prompt refinement is normal professional practice. Meta-prompting means asking GenAI to suggest better prompts. Meta-prompting can accelerate learning and retrieval-augmented approaches. Such as by providing GenAI with approved local documents or excerpts, and this process can improve contextual alignment, however the output will still require verification. ^12^ ^,^ ^18^

Table 1 shows a practical prompt framework (CRAFT) that can be taught quickly and reused across FD tasks. The CRAFT framework was developed by the authors. To support transfer, invite participants to save their best prompts securely to create a shared local prompt library that complies with institutional GenAIpolicy. Encourage participants to annotate prompts to indicate what worked, what failed, and what must be checked.

**Table 1.** CRAFT prompt literacy framework.

Principle	What it means in practice	Why it matters
C – Contextualise	Specify audience, setting, learning goals, constraints, and local norms; include what ‘good’ looks like.	Reduces generic outputs and improves alignment with your context.
R – Role-switch	Ask GenAI to respond from multiple perspectives (e.g., educator, learner, patient/care partner, different professions, skeptical reviewer).	Encourages critical thinking, reflection and assumptions to be explicit. Exposes power dynamics, and missing voices.
A – Augment (don’t automate)	Use GenAI to draft options, generate alternatives, or test ideas; keep humans accountable for final decisions.	Maintains human judgment, ethical responsibility, and contextual expertise.
F – Feedback-driven refinement	Iteratively improve prompts and outputs using explicit criteria; ask GenAI to critique its own output and suggest revisions.	Improves quality and helps participants build a habit of critical evaluation.
T – Traceability & ethics	Keep a prompt/output log; disclose use when appropriate; respect privacy, copyright, and sustainability constraints.	Supports transparency, governance, and continuous improvement.

*Vignette thread (Riverview - Step 3):* In week 1, the programme lead models CRAFT by live-prompting GenAI to draft a mini-workshop outline about feedback. Participants watch the ‘think-aloud’ process. First the audience is clarified: interprofessional clinical preceptors, who are asking for a bilingual handout draft. Next, a request for a skeptical reviewer critique is demonstrated followed by a discussion about how to revise the prompt to improve the output. Using participants then practice in pairs. GenAI prompt library as an example, participants are encouraged to contribute their improved prompts to a shared collection. ^19^

### #4. Co-create multilingual and context-specific resources using GenAI

FD programmes often need to work across languages, professional cultures, and local practice norms. GenAI can draft multilingual materials rapidly to produce items such as bilingual slides, facilitator guides, emails, and reflection prompts. GenAIcan also adapt materials to local practice contexts. This ability to translate materials is particularly useful when FD teams have limited translation or instructional design capacity. ^20^ ^,^ ^21^

However, translation and localisation are not the same. Even when a translation is grammatically correct, it may be culturally inappropriate, use unfamiliar professional terminology, or miss regional variation. For example, Spanish terms differ across countries and French terms differ between Québec and France. We recommend treating GenAI translations as drafts that require review by a local first-language speaker and/or subject-matter expert. For languages with limited GenAI support, including many Indigenous languages, human translation and community consultation are essential. ^15^

A helpful prompt strategy is to ask GenAI to: (a) propose two translation variants (formal vs conversational); (b) create a short glossary of key terms; (c) identify phrases that might carry unintended connotations; and (d) suggest how to maintain inclusive, non-stigmatising language in the target language. Regardless of tool (GenAI, Google Translate, DeepL, or other systems), accuracy and cultural safety should be verified before use.

*Vignette thread (Riverview - Step 4)*: The FD team drafts workshop materials in English, then uses GenAI to create a Spanish draft and a glossary of feedback terminology. Two bilingual educators review the draft, adjust region-specific terms, and flag a phrase that could be interpreted as blame toward learners. For an optional activity involving an Indigenous community scenario, the team consults a community liaison and does not use GenAI for translation.

### #5. Remix and adapt educational cases with GenAI to promote interprofessional learning and inclusion

Case-based learning is widely used in HPE and offers a natural entry point for GenAI-supported educational design. GenAI can help educators revise an existing case and create multiple versions. These versions can feature different professions in leading roles, different settings such as rural versus urban care, or different cultural or national contexts. GenAI can generate facilitator prompts, debrief questions, and assessment checklists. When used thoughtfully, GenAI can help FD programmes build learning materials that support more interprofessional and inclusive learning experiences. ^22^

Confidentiality is essential. Faculty developers should avoid entering real patient identifiers or identifiable learner information into public GenAI systems. Instead, use fictional or composite cases, and follow institutional and legal guidance on de-identification and data handling. In addition, GenAI-generated interprofessional details should be checked with local clinicians/educators from the relevant professions, not only with published literature because professional roles and scopes of practice vary across jurisdictions. ^14^

To reduce bias and improve realism, we recommend a structured representation and assumptions review. Table 2 provides example prompts/questions that faculty developers can use to audit cases for missing voices, stereotypes, power dynamics, resource assumptions, and stigmatising language. This review is best done with human co-reviewers, using GenAI as a support for generating alternative framings or identifying blind spots. ^14^ ^,^ ^15^ ^,^ ^20^

**Table 2.** GenAI-supported checklist to review cases for representation, assumptions, and potential harms.

What you are checking	Open-ended prompts/questions (to ask yourself, peers, and/or GenAI)
Representation and missing voices	Who is represented in this case, and who is missing (patient, care partner, nurse, pharmacist, community health worker, interpreter, etc.)? Who is portrayed as ‘default’?
Intersectional identities	How might intersecting identities (e.g., gender identity, race/ethnicity, language, disability, age, socioeconomic status) shape what happens in this scenario? What identities are assumed but not stated?
Power dynamics and roles	How are professional roles and decision-making power distributed? How could different professions take turns leading while others support? Are any professions portrayed as ‘helpers’ only?
Resource assumptions and access	What is being taken for granted about resources (time, staffing, technology, transport, insurance, supervision, interpreter access)? How might the case change in a low-resource or rural setting?
Language and framing	Are there value-laden or stigmatising words (e.g., ‘noncompliant’, ‘difficult’, ‘frequent flyer’)? What neutral, descriptive alternatives would be more appropriate (e.g., ‘not taking medication as prescribed’, ‘expressed frustration’, ‘frequent ED visits’)?
Stereotypes and narrative tropes	Does the case rely on stereotyped tropes or cultural superiority narratives? Can you provide an example of blame, heroism, or deficit framing—and how could it be rewritten?
Cultural safety and racism	Could any part of the case perpetuate racism or other forms of exclusion? What context is needed to avoid implying that inequities are caused by individuals rather than systems?
Disability and ableism	Are disability and accessibility needs portrayed respectfully? Are ‘unseen’ barriers considered (e.g., sensory impairment, neurodiversity, cognitive load)?
Realism and scope of practice	Does each profession’s role reflect local scope of practice and team workflows? What would local clinicians from each profession change?
Actionable revision	What specific change will we make now (change wording, add a character, adjust who speaks or acts, add context, change setting)?

*Vignette thread (Riverview - Step 5):* The FD team starts with an existing ‘difficult learner’ case and asks GenAI to generate three variants: (a) a community-based setting with limited resources; (b) a version where a pharmacist leads medication reconciliation; and (c) a version where a nurse leads the feedback conversation. Using Table 2, the team removes stigmatizing language, adds a care-partner perspective, and checks professional roles with local clinicians from each discipline.

### #6. Use GenAI to support feedback and mentorship

Feedback and coaching are central to FD, yet many educators struggle to make feedback specific, actionable, and aligned with learning goals. GenAI can support faculty developers by generating draft feedback phrasing, proposing alternative coaching questions, or helping educators anticipate learner reactions. Used well, GenAI can broaden the repertoire of feedback strategies available to faculty developers and participants. ^23^ ^–^ ^25^

A safe use pattern is to treat GenAI as a feedback rehearsal tool. For example, an educator can paste a de-identified excerpt of their written feedback (or a lesson plan) and promptGenAI to: (a) identify what is specific versus vague; (b) suggest more descriptive language; and (c) propose a next-step coaching question. To encourage reflection, use open-ended prompts such as: “Where is this feedback clear?” and “Where might feedback need more clarity or evidence?” Faculty developers should remind participants that GenAI suggestions may be inappropriate in certain cultural or supervisory contexts, therefore, output must be reviewed carefully as suggested in Applied Insight #4.

For higher-reliability outputs, consider retrieval-augmented workflows. Provide GenAI with approved local documents such as assessment policies, feedback models, accreditation standards and prompt it to align feedback suggestions with those documents. This process can reduce generic advice but does not eliminate the need for expert review. ^18^

*Vignette thread (Riverview - Step 6)*: Mid-programme, participants submit their draft workplace-based assessment plans. The faculty developer uses GenAI to generate coaching questions tailored to each plan and to draft examples of specific, behavior-based feedback statements. GenAI feedback is checked to ensure the language is inclusive and non-stigmatising by participants role-play feedback conversations to compare GenAI-suggested phrasing with their own language and discuss how certain phrases might be culturally inappropriate or overly supervisory.

### #7. Scaffold personalised professional development plans using GenAI

FD programmes often aim to support ongoing continuing professional development (CPD), yet participants may struggle to translate workshop insights into sustained change. GenAI can help participants draft and refine CPD plans by suggesting goals, milestones, resources, and self-monitoring strategies. ^26^ This approach can be aligned with self-determination theory by supporting autonomy, when participants choose goals, competence, with plans that include achievable milestones, and relatedness, if plans include peer or mentor support. ^27^ ^,^ ^28^

A practical workflow begins with inviting participants to draft their own CPD goals. The goals can be used to prompt GenAI to propose alternative ways to frame goals and milestones. The GenAI output can be used to refine the plan with a mentor/coach, ensuring alignment with institutional expectations, accreditation and regulatory standards, and local teaching realities. Participants can be encouraged to track their progress using simple tools such as a learning management system discussion thread, a shared spreadsheet, or a project board such as Trello.

Faculty developers should anticipate that participants will differ in their access and skill with GenAl. This inequity may reflect differences in tool accessibility, time available to learn, differences in language proficiency, or comfort with GenAI. Mitigation strategies include offering low-tech alternatives, sharing institutionally supported tools, providing prompt templates, and creating psychologically safe spaces to discuss failures and uncertainties. ^29^ Examples of tools with different access models and practical applications are summarized in Table 3.

**Table 3.** Examples of tools and resources that may support GenAI-enabled faculty development (non-exhaustive; features and cost models vary).

Category	Examples (illustrative)	Typical access model	Notes for faculty developers
LLM chat assistants	ChatGPT, Claude, Gemini, Copilot	Free tiers and paid tiers; enterprise options may be licensed by institutions	Prefer institutionally approved tools for any work involving internal documents; verify outputs.
Institutional copilots	Microsoft Copilot (institutional), other integrated copilots	Typically institution-licensed	May offer stronger privacy controls; confirm local settings and policies.
AI search with citations	Perplexity, Elicit, Consensus, Open Evidence	Free/paid tiers	Can speed up literature scans, but still verify sources; beware fabricated citations.
Translation support	DeepL, Google Translate, GenAI translation workflows	Free/paid tiers	Treat as draft; review with local first-language speaker; consider cultural safety.
Prompt libraries/templates	Maastricht University AI Prompt Library; local prompt libraries	Often free/open; local libraries may be internal	Use to scaffold prompt literacy; include disclaimers and verification steps.
Project management/tracking	Trello, Planner, Notion, shared spreadsheets	Free/paid tiers	Useful for tracking CPD plans and QI cycles; avoid storing sensitive data without approval.
Quality and inclusion review	Inclusive language checkers; structured checklists; peer review groups	Varies	Automated checkers can help but should not replace human co-review, especially for local cultural context.

*Vignette thread (Riverview - Step 7):* In week 9, each participant drafts a CPD plan focused on improving feedback in their setting. GenAI suggests milestones (e.g., ‘pilot a new feedback tool in two clinical shifts’), prompts participants to identify peer support, and flags misalignment with an accreditation requirement. Participants review plans with a mentor and choose a tracking method; the FD team also provides a non-GenAI template for participants who cannot or prefer not to use GenAI.

### #8. Evaluate continuously and iteratively

GenAI integration should be evaluated as part of the FD programme, not treated as a one-time implementation decision. ^30^ Evaluation should move beyond measuring traditional outcomes such as satisfaction, learning, and behaviour change. Evaluation should assess the accuracy and quality of GenAI-assisted outputs, collect data about participant experiences of equity and inclusion, describe unintended consequences, such as over-reliance on GenAI, reduced critical thinking, or widened gaps between those with and without GenAI access. Evaluation should also provide information about sustainability implications such as time, cost, and environmental impact.

A simple continuous quality improvement approach involves treating GenAI use as a set of testable changes. Plan–do–study–act cycles can be used to trial a prompt library, a checklist, or a new GenAI-supported workflow, then the outputs can be refined based on feedback. Faculty developers can also collect ‘near-miss’ stories such as the times GenAI produced plausible but wrong or harmful output, as learning tools to strengthen critical evaluation.

Priority research questions emerge from these insights. Questions include: Which prompt literacy teaching strategies most improve educator confidence and critical evaluation? What governance approaches best reduce privacy and equity harms? How does GenAI use affect educator workload and quality of outputs over time? And what evaluation measures can capture both benefits and risks of GenAI-supported FD in diverse HPE contexts?

*Vignette thread (Riverview - Step 8):* After the programme, the FD team uses GenAI to summarise de-identified reflection comments and to generate possible improvement actions. The team also reviews several ‘near-miss’ examples where GenAI output was based on inappropriate assumptions about resources in rural sites. The team revises their checklist, updates the prompt library with stronger context prompts, and adds an explicit session on equity and data sovereignty for the next cohort.

## Conclusion

GenAI is reshaping how educational materials and professional learning can be designed and delivered. For faculty developers, the most productive stance is neither uncritical adoption nor blanket avoidance, but intentional integration with explicit governance, modelling of prompt literacy, and rigorous human oversight.

The eight applied insights presented here are intended to be adapted to local contexts. They emphasise that the faculty developer’s, and broader educator communities’ judgment, ethics, and contextual understanding remain central, even as GenAI tools become more capable. The longitudinal vignette illustrates how these insights can be applied as a coherent workflow across FD phases.

Responsible GenAI use in FD requires collaboration with multiple interest holders, including learners, patients/care partners (when cases reflect lived experience), institutional leaders, information security teams, and communities affected by the educational content we create. By embedding equity, confidentiality, and sustainability considerations early, faculty developers can help ensure GenAI supports, rather than undermines, high-quality, inclusive interprofessional development.

## Data availability

No datasets were generated or analysed for this applied insights article. The vignette is fictional and presented for illustrative purposes only.
