# Supplementary material for: Applied insights for using Generative Artificial Intelligence in Faculty Development in Health Professions Education
Source: MedEdPublish (2016). 2026 Jun 15;15:279. Originally published 2025 Dec 8. [Version 2] doi: 10.12688/mep.21403.2 (PMC12811717; doi:10.12688/mep.21403.2)
Supplement: Peer review history

# Reviewer report: Reviewer response for version 2

**DOI:** 10.21956/mep.23231.r46062

**Published:** 2026-07-07

**Authors:** Jennifer Benjamin

**Affiliations:** 1 Baylor College of Medicine, Houston, Texas, USA

No further changes

If evidence from practice is presented, are all the underlying source data available to ensure full reproducibility?

Yes

Is the topic of the practical tips discussed accurately in the context of the current literature

Yes

Are all factual statements correct and adequately supported by citations?

Partly

Are the conclusions drawn balanced and justified on the basis of the presented arguments?

Yes

Are arguments sufficiently supported by evidence from the published literature and/or the authors’ practice?

Yes

Reviewer Expertise:

AI, Faculty Development

I confirm that I have read this submission and believe that I have an appropriate level of expertise to confirm that it is of an acceptable scientific standard.

# Reviewer report: Reviewer response for version 2

**DOI:** 10.21956/mep.23231.r46059

**Published:** 2026-06-17

**Authors:** Zubin Austin

**Affiliations:** 1 University of Toronto, Toronto, Ontario, Canada

The authors have effectively addressed my concerns and recommendations in this revision and I am pleased  to  recommend acceptance and publication of this work.

If evidence from practice is presented, are all the underlying source data available to ensure full reproducibility?

Yes

Is the topic of the practical tips discussed accurately in the context of the current literature

Yes

Are all factual statements correct and adequately supported by citations?

Yes

Are the conclusions drawn balanced and justified on the basis of the presented arguments?

Yes

Are arguments sufficiently supported by evidence from the published literature and/or the authors’ practice?

Yes

Reviewer Expertise:

Health professions education

I confirm that I have read this submission and believe that I have an appropriate level of expertise to confirm that it is of an acceptable scientific standard.

# Reviewer report: Reviewer response for version 1

**DOI:** 10.21956/mep.22923.r44831

**Published:** 2026-02-04

**Authors:** Ming-Ka Chan

**Affiliations:** 1 University of Manitoba, Manitoba, Canada

Thank you for the opportunity to review. I found the article of interest as a faculty developer - it has many pragmatic tips.

 I made some suggestions below and happy to share the comments as embedded within the manuscript if that would be helpful. I think if some areas are further clarified or elaborated with concrete examples, this paper could be even more useful. As always scaffolding with a conceptual framework would strengthen the paper: Zhou, X., & Schofield, L. (2024). Developing a conceptual framework for Artificial Intelligence (AI) literacy in higher education. *Journal of Learning Development in Higher Education* could be one example. There are several AI-specific frameworks to consider. Consider providing further evidence for some of your recommendations e.g. do you have a reference re ‘faculty who demonstrate effective prompting help peer build confidence’.

 I would have liked some reference to the other considerations that one needs to have whenever  using AI - for example, what is the impact of the use of GenAI on our water resources and planet's health in general. The responsible use of AI needs to be one of the early questions. What about the ethical aspects - data sovereignty for example (see CARE-AI Framework - Equity in Health Systems for more details about the ethics in AI application in the health and health education context). As educators, we have a role to play in looking at the big picture and determining how the changes we implement may affect other domains and aspects of education, work and society.

 1. Language - there is use of language that is not clear to me for example (and see list within specific sections of the paper: 

 a) Staff members as terminology is unclear although examples are given. I wasn't expecting this list. I wonder instead about the use of faculty members instead given we are talking about faculty development. Unless this term is not used in all health professions?

 b) Consistency of terminology - e.g. student - perhaps 'learner' would be the broadest term and is used in the rest of the document.

 c) imbalanced language - what does this mean?

 d) cliche is not the same as a stereotype - instead may want to use the term bias (which was done throughout the rest of the document.

 2. There are several resources or platforms mentioned however it is not clear if these are require payment. A table outlining how they would be useful would add to the paper. And any challenges in using them as certainly factors like cost impact access.

 I add in my other comments based on the respective section within the paper:

 Applied insight #1

 - Suggest use of HPE throughout (used med ed in this section)

 - ‘An overarching theme is that the human remains the ultimate decision-maker. Faculty developers need to consider if the data will be used to train the GenAI tool, construct new models, or to generate new content for other users.’  - these two sentences aren't fully connected. A sentence to link would be helpful.

 - Local Institutional guidelines - I think there may be other bodies beside local that may have guidelines so perhaps 'local and other national or international guidelines'?

 - Biases: Would add to this list, biases based on racism and other forms of exclusion. It is more than stereotyping or underrepresentation. It is about the images used: tendency to be healthy young adults with normal BMI without disability. Children or Older adults are rarely portrayed. It is about pronunciation of names with only English linguistic sounds - consider google maps cannot pronounce French named streets in Canada which has two official languages - English and French.

 - There also needs to be active training of the GenAI models to address the data. In the latter case, having it use French names in its data sets. It is also about the words we use e.g. stakeholder is colonial and could be replaced with interest holder, crazy reflects a bias towards those with mental health needs or the use of black to have negative connotations

 - You list several AI platforms – this may be perceived as a potential conflict unless you can explain why these platforms are better than others. Perhaps a table sharing the nuances of each of these platforms might be helpful

 - May want to elaborate about what is meant by sensitive data. The novice reader may not understand the concept

 - The bias pass mentions the representation audit but then there are many other elements in the paragraph - Would add more components. Or reword as bias pass could start with a representation audit.

 - Suggest more than one reviewer. In Canada, would suggest having members of First Nations, Metis and Inuit groups be part of the review. The bias pass also has to consider aspects like data sovereignty if you are inputting data into the model.

 - Suggest that this review is more than about sense making. Considering output from different perspectives and review of language that might harm one or more groups etc

 - I found the use of ‘tone in writing’ here to be interesting. I might be broader and state that we are asking the reviewer to spot biases that we may have missed (including use of language and images)

 - Are we meaning QA for accreditation purposes or CQI (therefore the term would be QI) or both?

 Table 1

 - Role switching: What about participants or learners? And why framed as 'expert roles'

 Appendix

 - ‘  Would this be fair and inclusive in my institution’s context?’ - What is 'this' referring to? Outputs?

 -‘ If using references, make sure you check them all and that these are being used appropriately.’ Would be great if a suggestion was provided about how to do this i.e. when multiple references that one might not be familiar with.

 - example of vague term and replacement would be helpful here.

 - ‘Suggest how to phrase it to get authoritative yet supportive responses.’ – example would be helpful here.

 Applied Insight #4

 - You mention Spanish as another language – perhaps you may just want to say another language and may be important to give some caveats here. For example, Gen AI can only translate a few Indigenous languages and often there are limitations e.g., accuracy and cultural context

 - There are some negative connotations with term 'native' – ‘first-language’ speaker may be a good alternative. Also wonder about adding in concept of local first-language speaker. e.g. French in Quebec is different from French in France

 - Not sure the literature will necessarily be helpful with identifying roles and scope of practice. The roles and scope of practice also vary globally and it would be important to consult local health professionals to better understand the real life local context

 Table 2

 - Not fully clear what unequal roles means.

 - I like how the first question is open-ended and wonder if the rest of the questions in this table could also be open-ended. E.g/, How could different professions take turns in a leading role while others support? What patterns are you noticing?

 - I have been taught that invisible is an ableist term. Perhaps unseen?

 - 'Would the case still “work” if we changed the gender, ethnicity, or socioeconomic status of the main character.‘ Perhaps you could add 'or other identity' of the main character

 - ‘particular identity’ - Given we all have multiple intersecting identities, perhaps make this plural.

 - blame, heroism, or cultural superiority: Would be helpful to see an example of each of these.

 - RE: To reveal assumptions (e.g. that all learners have the same resources,: Is it about having the same resources or similar access to resources? Also this example doesn't align with the question to the left as currently listed. Would suggest including learners in your list as not all teams have learners in all settings.

 - RE: What is being taken for granted about this patient, team, or setting?: Would add care partners here in this list...aka pediatrics is dependent on care partners for care of the infant/child and perhaps less so for the adolescent. Similar for many geriatric cases.

 - RE: Are there value-laden words (e.g. “difficult”, “demanding”, “noncompliant”) that could be replaced with more neutral descriptions?: would be great to see the replacement term here

 - RE: more descriptive, less judgmental language: May want to add in: ‘More context is provided.’

 - What perspective might be missing (patient, family, nurse, pharmacist, community)? – consider changing family to care partner given that supports may not be family in the ‘legal’ sense of the word

 - RE: To remind faculty to add voices that are often overlooked but relevant to real practice.- I try to avoid the use of 'but', often substituting with ‘and’.  In this case, I think it is about 'despite' being relevant to real practice.

 - RE: What specific change will I make now (e.g. change wording, add a character, adjust who speaks)? Consider ‘speaks or acts?’

 - Applied insight #1 reference: Initially I was not sure what this is referring to and wondered if it was the first example in this table. Suggest you write in the text for applied insight #1 and then refer to it for more details aka: Ground your approach to using GenAI in faculty development with key principles (applied insights #1)

 Applied Insight #6:

 - “Do you feel your assessment plan is clear? - Suggest reframing as an open ended question e.g., Where do you feel your assessment plan is clear and where are the areas that are less clear?

 Applied Insight #7

 - Self determination theory: You mention autonomy and I feel like there could be opportunity to connect to the other components I.e. competency and to relatedness. In fact so many of the recommendations involve feedback seeking from peers, experts, interest holders etc  it naturally builds relatedness.

 - …refine and customize to meet regulatory standards and supervisor expectations: I feel like a broader term for supervisor might be useful here...teacher expectations perhaps. Supervisor seems limited. I think regulatory and accreditation standards are different so perhaps list both in this sentence?

 - Peer ideas: ideas from peer and mentors? I think there is a nuance here that is different.

 - tracked using platforms such as TrelloMay want to have a table for quick reference to all the tools that can be used to support integration of Gen AI. Also can you elaborate a bit more what this tracking program is about to give the reader more contextApplied Insight #7

 - Not sure what unequal use of GenAI means? Is it inequitable access to GenAI? Or ability to use GenAI?

 Conclusion

 - …no matter how sophisticated GenAI tools become, it is the faculty developer’s judgment, ethics, and contextual understanding: In addition to faculty developers, consider adding other interest holders...especially given the multiple points of consultation.

 - faculty developers offer more inclusive interprofessional development - the way this sentence reads is that the inclusive element is about interprofessional.... There are many elements of inclusivity.

If evidence from practice is presented, are all the underlying source data available to ensure full reproducibility?

Yes

Is the topic of the practical tips discussed accurately in the context of the current literature

Yes

Are all factual statements correct and adequately supported by citations?

Partly

Are the conclusions drawn balanced and justified on the basis of the presented arguments?

Yes

Are arguments sufficiently supported by evidence from the published literature and/or the authors’ practice?

Yes

Reviewer Expertise:

social justice, leadership education, competency-based education, program evaluation

I confirm that I have read this submission and believe that I have an appropriate level of expertise to confirm that it is of an acceptable scientific standard, however I have significant reservations, as outlined above.

# Reviewer report: Reviewer response for version 1

**DOI:** 10.21956/mep.22923.r44968

**Published:** 2026-01-16

**Authors:** Jennifer Benjamin

**Affiliations:** 1 Baylor College of Medicine, Houston, Texas, USA

The manuscript is timely, well written, and reimagines the conceptual nuances of GenAI usage. It makes a clear contribution to the evolving literature on GenAI in faculty development (FD) by shifting the focus from *teaching about AI* to *using AI as a co-developer*. The authors adopt a novel approach of deriving insights from a workshop to inform applied  insights. The following are suggestions to improve the quality of the submission.

 Introduction:

1. Was IRB approval obtained to write up this workshop?
2. Paragraph 2 2nd line: AI generating content without compromising quality, needs to be elaborated more as most of AI outputs need human oversight. This statement may be overstating the capability of some of AI tools, which is misleading. Is there a reference to support this statement?  
3. Paragraph 3 Line 2:  statement about’ Faculty developers must manage questions of accuracy, privacy, and fairness’ clarify what managing fairness means, do you refer to faculty needing to ensure accuracy or address concerns re these characteristics
4. While the manuscript references multiple pedagogical concepts (human-in-the-loop, cognitive apprenticeship, self-determination theory), it does not clearly articulate a unifying conceptual framework. It might be helpful to consider entangled pedagogy to consider how faculty and learners co-create these solutions.
5. #1Paragraph 3 how about Co-pilot? It might suffice to just state institutional AI tools. All of these tools mentioned such as biasly, perplexity might need some definitions on their functionality.
6. Provide context to biasly, the link is a website that does not demonstrate AI assessment of language.
7. Explicitly anchor the insights to **one or two recognizable frameworks** (e.g., faculty development, educational design research, or established AI-in-education models).
8. #3 The current models compensate for badly written prompts and meta prompting can be used to improve the functionality pf LLMs with complex tasks. That said, it also worthwhile noting that outputs can be refined using iterative prompting andusing RAG, both of which can be elaborated on further.
9. **Strengthen the “Evidence from Practice” Claim**
  The manuscript states that insights are informed by workshop discussions and lived experience, but these data are largely implicit. Improve the arguments to include concrete facts.
10. Include statements such as AI generated content is not the final product and educators should be responsible with AI use
11. # 3 teach prompt literacy: Line 1 core? Instead of central?
12. Is there a citation for this claim that faculty who demonstrate effective prompting help peer build confidence
13. The mastrick university link does not work
14. #4Paragraph 2 Has the accuracy of these tools you are suggesting been studied?
15. How about google translate?
16. Last line: change to need to double check for accuracy, instead of may need to invite native speaker
17. Opportunity for Integrative Case or Thread
18. Issue: Each insight stands well on its own, but the manuscript reads as a collection rather than a cohesive applied narrative.
19. Consider adding a single longitudinal vignette (e.g., an interprofessional FD program evolving over time) revisited briefly across several insights. This would improve narrative coherence and reduce cognitive load for readers.
20. #6 Using RAG might improve AI performance to provide robust feedback
21. Terminology such as “prompt literacy” and “GenAI checklist” could benefit from brief operational definitions at first use.
22. The conclusion is strong but could be improved by identifying priority research questions emerging from these insights.
23. Ensure consistency in capitalization and hyphenation of “GenAI” throughout
24. This is a valuable, timely, and well-constructed applied article,  it would benefit from sharpening its scholarly scaffolding around an already strong practical core.

If evidence from practice is presented, are all the underlying source data available to ensure full reproducibility?

Yes

Is the topic of the practical tips discussed accurately in the context of the current literature

Yes

Are all factual statements correct and adequately supported by citations?

Partly

Are the conclusions drawn balanced and justified on the basis of the presented arguments?

Yes

Are arguments sufficiently supported by evidence from the published literature and/or the authors’ practice?

Yes

Reviewer Expertise:

AI, Faculty Development

I confirm that I have read this submission and believe that I have an appropriate level of expertise to confirm that it is of an acceptable scientific standard, however I have significant reservations, as outlined above.

# Reviewer report: Reviewer response for version 1

**DOI:** 10.21956/mep.22923.r44966

**Published:** 2026-01-05

**Authors:** Komal Srinivasa

**Affiliations:** 1 University of Auckland, Auckland, New Zealand

This article uses eight insights to provide practical guidance on how to integrate GenAI for faculty development. The tables used for prompt literacy are practical and helpful, and the questions to check for representation and assumptions add strength to this. In the section on remixing and adapting education cases, the addition of a statement on maintaining patient confidentiality and other institutional GenAI guidance would be beneficial.

If evidence from practice is presented, are all the underlying source data available to ensure full reproducibility?

Not applicable

Is the topic of the practical tips discussed accurately in the context of the current literature

Yes

Are all factual statements correct and adequately supported by citations?

Yes

Are the conclusions drawn balanced and justified on the basis of the presented arguments?

Yes

Are arguments sufficiently supported by evidence from the published literature and/or the authors’ practice?

Yes

Reviewer Expertise:

Quality assessment, online videos, e-learning, rubric development

I confirm that I have read this submission and believe that I have an appropriate level of expertise to confirm that it is of an acceptable scientific standard.

# Reviewer report: Reviewer response for version 1

**DOI:** 10.21956/mep.22923.r44836

**Published:** 2025-12-29

**Authors:** Zubin Austin

**Affiliations:** 1 University of Toronto, Toronto, Ontario, Canada

This is an interesting and well written article addressing a topic of timely importance.  I have not seen other authors tackling this important issue, so I applaud the authors for their initiative in addressing the issue.

 Overall I found the hints and tips provided to be practical, useful, and generally well explained. I believe the article will be of value to many readers.  I believe the article could be enhanced however, if there were some additional practical examples of how each of the tips could be applied in a real world setting.  To this end, I would suggest the authors consider incorporating some kind of a longitudinal case study in the article. Provide a brief context relevant to faculty development (ideally one that is interprofessional or at least relevant to fields outside medicine).  With that, for each of the tips presented, provide a brief scenario where that tip is applied in the context of that case.  This would really help readers bring the content to life in a practical manner.  While the authors have indeed been practical in their approach, as currently written I do not believe the article lives up to its fullest potential as the application piece requires some work by readers to visualize...if the authors could support this visualization through a case study embedded in the article, I believe the piece would be much enhanced.

 The specific insights offered by the authors are very helpful, particularly those related to prompt engineering and needs assessment.  These insights are really useful to share in the format they have elected to do so. Overall I enjoyed reading this insightful work and hope the author are able to conceptualize a relevant case study to enhance applicability of their insights.

If evidence from practice is presented, are all the underlying source data available to ensure full reproducibility?

Yes

Is the topic of the practical tips discussed accurately in the context of the current literature

Yes

Are all factual statements correct and adequately supported by citations?

Yes

Are the conclusions drawn balanced and justified on the basis of the presented arguments?

Yes

Are arguments sufficiently supported by evidence from the published literature and/or the authors’ practice?

Yes

Reviewer Expertise:

Health professions education

I confirm that I have read this submission and believe that I have an appropriate level of expertise to confirm that it is of an acceptable scientific standard, however I have significant reservations, as outlined above.
